# Optical Dark Rogue Wave

**DOI:** 10.1038/srep20785

**Published:** 2016-02-11

**Authors:** Benoit Frisquet, Bertrand Kibler, Philippe Morin, Fabio Baronio, Matteo Conforti, Guy Millot, Stefan Wabnitz

**Affiliations:** 1 Laboratoire Interdisciplinaire Carnot de Bourgogne (ICB), UMR 6303 CNRS - Université de Bourgogne Franche-Comté, F-21078 Dijon, France; 2 Department of Information Engineering, Università di Brescia, and INO-CNR, Brescia, Italy; 3 Univ. Lille, CNRS, UMR8523 - PhLAM - Physique des Lasers Atomes et Molécules, F-59000 Lille, France

## Abstract

Photonics enables to develop simple lab experiments that mimic water rogue wave generation phenomena, as well as relativistic gravitational effects such as event horizons, gravitational lensing and Hawking radiation. The basis for analog gravity experiments is light propagation through an effective moving medium obtained via the nonlinear response of the material. So far, analogue gravity kinematics was reproduced in scalar optical wave propagation test models. Multimode and spatiotemporal nonlinear interactions exhibit a rich spectrum of excitations, which may substantially expand the range of rogue wave phenomena, and lead to novel space-time analogies, for example with multi-particle interactions. By injecting two colliding and modulated pumps with orthogonal states of polarization in a randomly birefringent telecommunication optical fiber, we provide the first experimental demonstration of an optical dark rogue wave. We also introduce the concept of multi-component analog gravity, whereby localized spatiotemporal horizons are associated with the dark rogue wave solution of the two-component nonlinear Schrödinger system.

During the last couple of decades, the study or rogue waves (also known as freak waves), which are high amplitude surface waves in open water, has attracted a great deal of attention since the first scientific measurement, by means of a laser beam, of an exceptionally large “Draupner wave” at an oil platform in the north sea on new year’s day of 1995^1^. Although rogue waves are generally referred to as giant waves of exceptional elevation, it is known to sailors that rogue waves are often accompanied by deep troughs or, as the nautical lore goes, “holes in the sea”. In spite of their extensive studies, the mechanisms at the origin of rogue waves remain to date largely unexplained. Yet, it is known that big waves form with greater regularity in ocean regions swept by powerful currents, e.g., the Agulhas around South Africa, the Kuroshio off Japan, the Gulf Stream off the eastern United States and in the famous Bermuda triangle. In these waters, surface waves meet currents running in the opposite direction. As a matter of fact, scientists who examined satellite images of the Agulhas current in the Indian Ocean recently revealed the formation of “eddies”, or transient coherent whirlpools that remain intact for months^2^. These rogue waves have been renamed as oceanic black holes, since they represent the black holes of turbulence: they are fully analog to photonic gravitational black holes^2^.

Quite remarkably, significant progress in our understanding of rogue wave formation has been enabled in recent years by simple photonic test bench emulators, thanks to analog models based on laser light propagation in optical fibers^3^,^4^,^5^. So far, optical rogue wave experiments have demonstrated different mechanisms for the generation of large amplitude transient optical waves, such as for example the Peregrine soliton^6^. In this work, we theoretically and experimentally consider the optical analog of crossing sea states, which is described by means of the coupled nonlinear Schrödinger equations (NLSEs)^7^,^8^. We provide what is, to our knowledge, the first experimental demonstration, by using standard telecom components and fiber, of the generation of an optical dark rogue wave (ODRW), which is a transient hole of light where the optical intensity is almost zero at its nadir.

The interest of our study extends beyond the field of rogue wave phenomena, since it permits us to speculate an intriguing connection between optical rogue waves in fibers and analogue gravity models. In recent years, technological advances in the field of photonics have stimulated researchers to develop a few analogues of general relativistic gravitational phenomena within compact lab benches, thus evidencing observations of event horizons, gravitational lensing and Hawking radiation^9^,^10^,^11^,^12^,^13^,^14^. With the aim of gaining new insights into inaccessible astronomical objects, the implementation of such powerful tools remains a major challenge when studying the coupling between different gravitational features^15^. Previous experiments were based on the observation of laser light propagation through an effective moving medium or space flow, which is obtained by exploiting the intensity-dependent refractive index or Kerr effect, for example induced by a co-propagating optical pulse.

In fact, a high-power femtosecond optical pulse was previously launched in the anomalous dispersion regime of a photonic crystal fiber to form an optical soliton^10^. Such pulse generated, via cross-phase modulation (XPM), a time-varying refractive index shift, hence providing an effective moving medium for the propagation of a frequency tunable quasi-continuous wave (cw) probe that was injected at a widely different wavelength in the normal dispersion regime of the fiber. Dispersion engineering permits to match the group velocities of the soliton pulse and the probe, so that the probe is slightly slower (or faster) than the pulse. When a collision with the leading edge (trailing edge) of the pulse occurs, via XPM the cw probe experiences a frequency shift towards the red (blue). This leads to probe acceleration (slowing down), until a full matching of the pulse and probe group velocities is achieved. This corresponds to reaching “points of no return” – probe light cannot cross the pulse and an event horizon is thus realized. Although purely classical, such effect can be interpreted as mimicking the kinematics of light at a black hole or a white hole optical event horizon, respectively^10^,^12^ (see Fig. 1b,c). Moreover, the collision between optical solitons and dispersive waves at event horizons has also been recently proposed as a possible mechanism for the generation of rogue wave events^16^,^17^.

A complementary and fully equivalent description of the temporal collision between the soliton pulse and the weak probe may be provided in the frequency domain. The permanent red and blue frequency shifts of the probe that occur at the black and white hole horizons correspond to the frequency conversion of the probe signal into an idler^14^. Recent experiments have further demonstrated that it is not necessary to use high power femtosecond pulse sources to induce the time localized refractive index perturbations that provide the effective flowing medium for the probe^14^. In fact, a mode-locked short pulse laser source emits a frequency comb in the Fourier domain. An equivalent frequency comb may be dynamically generated along the fiber via cascaded four-wave mixing, by simply launching two intense cw pumps at the fiber input^14^. In this way analog event horizon phenomena can be reproduced by means of standard fiber optics telecom equipment.

So far, analogue gravity kinematics was limited to scalar optical wave propagation test models. On the other hand, there is currently much interest in the study of multimode nonlinear optical effects^18^. Complex nonlinear mode coupling interactions exhibit a much richer spectrum of excitations with respect to the scalar case, thus potentially expanding the range of space-time analogies, for example to the case of multi-particle interactions and quantum gravity^19^.

In this work, we introduce the concept of multi-component analog gravity, by exploiting the nonlinear polarization interaction of light beams in a randomly birefringent telecommunication optical fiber. We point out that the generation of spatiotemporal localized event horizons is associated with the sudden emergence (and disappearance) of the ODRW solution of the coupled NLSEs. Our results may provide important physical insights in the phenomenology of other implementations of analog gravity models, e.g., in condensed matter physics and hydrodynamics^19^,^20^,^21^,^22^,^23^,^24^,^25^.

To gain a simple intuition of the physical mechanism leading to multicomponent horizons in optical fibers, let us consider the following reasoning. As it is well known, if a light pulse propagates through a normally dispersive medium, its higher frequency components travel slower than its lower frequency components. Therefore the instantaneous frequency grows larger with time across the pulse profile. Moreover, if two pulses are injected in the fiber with different carrier frequencies, the high frequency pulse will travel with a slower group velocity with respect to the low frequency pulse. Suppose that the two frequency-shifted pulses are injected along orthogonal state of polarizations (SOPs). Optical fibers have the remarkable property that, if the relative frequency shift is sufficiently low, the two pulses maintain well their orthogonality along the entire propagation length, even though the local fiber birefringence varies randomly along the propagation distance and in time. In a spatially rotating frame of reference such that the fast linear evolution of the SOP along the fiber is removed, the two pulses represent linearly uncoupled propagation channels, where light moves at different speeds.

When using a reference frame travelling with the average of the velocities of the two polarization channels, the situation is completely analogous to the flow of water in two oppositely directed currents^7^,^8^. Now as the optical intensity of the pulses grows larger, the optical Kerr effect leads to a power-dependent contribution to the fiber refractive index. This induces an instantaneous frequency shift or chirp across the intensity profile of the pulses, hence, in combination with dispersion, to a temporal advance or retardation of the pulse fronts with respect to the average group velocity (which is zero in the travelling frame). Ultimately, the interplay of the Kerr effect and dispersion forces the light to stop at well-defined event horizons that surround a space-time localized ODRW as summarized in Fig. 1. In hydrodynamics, this situation corresponds to the growth of the crest or the deepening of the trough of a mass of water, as a result of a non-uniform water wave velocity profile and nonlinearity^7^,^8^. Quite remarkably, the nonlinear fiber optics situation is also completely analogous to the propagation of two-component Bose-Einstein condensates, which exhibit black or white hole horizon phenomena^19^,^20^,^21^,^22^,^23^.

Note that, in contrast with earlier fiber optics experiments on analog event horizons^10^,^14^, in our case there is no soliton-induced black hole or white hole horizon experienced by a probe pulse. On the contrary, a dark rogue wave is formed from the polarization modulation instability (PMI) of the two input orthogonally polarized and frequency shifted pumps. Yet, quite strikingly, we find that the ODRW may be associated with the presence of ephemeral (or spatiotemporal localized) event horizons.

## Results

### Two-component Manakov system

The growth of temporal patterns such as bright solitons^26^ and rogue waves^3^,^4^,^5^,^6^ from an initial quasi-cw background is activated in optical fibers by the MI process. In the normal dispersion regime (which corresponds to the shallow water regime in hydrodynamics), there is no MI for scalar waves. However the nonlinear wave coupling between two orthogonal polarization modes may still lead to the MI of an intense cw field. In telecommunication fibers with a random rotation of the local birefringence axes, it is possible to average out the short-scale linear polarization oscillations in order to describe nonlinear pulse propagation effects^27^. This leads to the integrable, incoherently coupled NLSEs, also known as Manakov equations^28^. The propagation of two orthogonally polarized optical pumps at a relative frequency offset, say, Δ = Δω/2π, is described by the two-component Manakov system


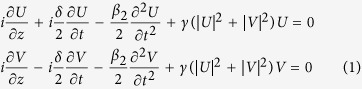


**Figure 1:**
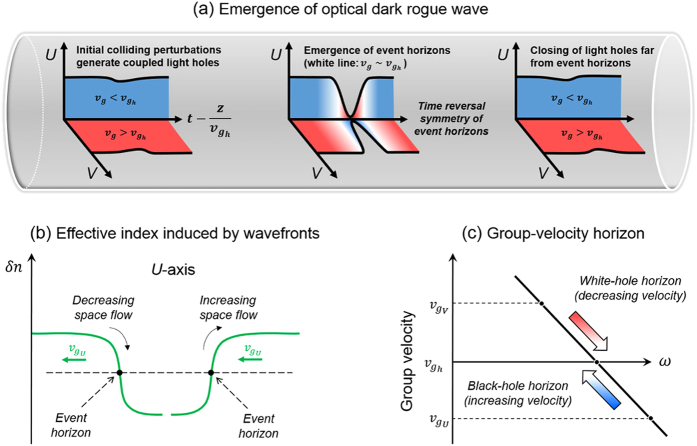
Illustration of the optical ODRW solution of the vector nonlinear fiber-analog system. (**a**) Evolution of the temporal wave profiles in the two propagation channels (orthogonal polarizations U and V) of the optical fiber, plotted in a reference frame moving with the group velocity at horizon. (**b**) Event horizons generated in the propagation channel U in analogy to the flowing river of space in astrophysical black and white hole horizons^12^. The gradient of the gravitational field across the horizons is analogous to the gradient of the refractive index induced by orthogonally polarized modulated wavefronts. (**c**) Corresponding laboratory-frame group velocity plotted as a function of frequency, channels U and V exhibit linear group velocities with opposite signs relative to the effective group velocity at horizons.

Here z and t denote the propagation distance and retarded time (in a frame travelling at the group-velocity evaluated at the central carrier frequency ω_o_) coordinates; *U* and *V* are the complex slowly varying amplitudes of the two pump waves at frequencies ω_u,v_ = ω_o_ ± πΔ, respectively, and d =*β*_*2*_ D is associated with their group-velocity mismatch (GVM) owing to normal group-velocity dispersion. In fact, the U (V) pump is a slow (fast) wave with respect to waves at the carrier frequency ω_o_. *β*_*2*_ and *γ* are the group-velocity dispersion and the effective Kerr nonlinear coefficient at frequency ω_o_, respectively. As discussed in the Supplementary Section, whenever two cws with different carrier frequencies and orthogonal SOPs are injected in a fiber, they are subject to polarization MI (PMI)^29^. As we shall demonstrate next, the evolution of PMI may lead to the creation of an ODRW (see Fig. 1a).

### Polarization rogue wave

**Figure 2:**
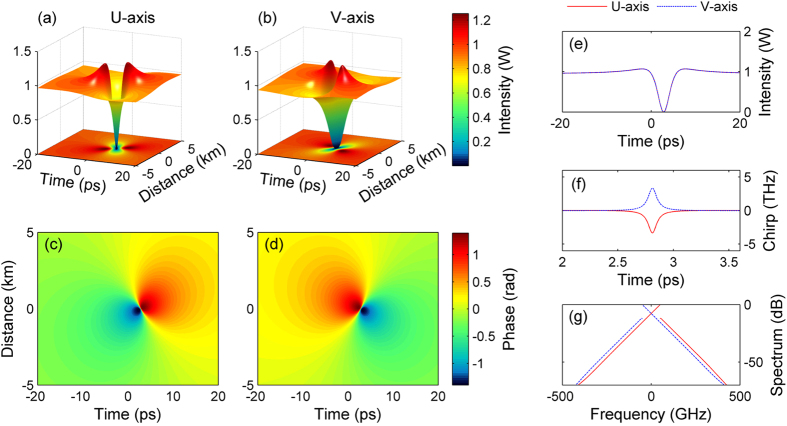
ODRW solution of the vector NLSE in the normal dispersion regime. (**a,b**) 3D and contour plots showing the dependence of optical power on time and propagation distance in the U and V polarization modes (note that the distance of the light hole center is arbitrarily shifted at the origin). (**c,d**) Contour plots of the phase in both polarization modes. (**e**) Time dependence of power in each polarization at z = 0, showing that zero intensity is reached at t near 2.8 ps. (**f**) Associated instantaneous chirp or frequency shift with opposite sign in each polarization. (**g**) Corresponding asymmetric power spectrum of the field in each of the two polarization modes.

The variation of optical power and phase with time and space coordinates for the two orthogonally polarized, frequency-shifted waves composing an ODRW solution of the Manakov system is illustrated in Fig. 2. As can be seen, the power of both polarization components exhibits a sudden and isolated dip all the way down to almost zero (see panels (a-d)). Dark rogue wave solutions have a simple analytic representation as the ratio between two polynomials (see Supplementary Section), which can be obtained by the Darboux dressing method based on the inverse scattering transform formalism^29^. Panels (e-g) in Fig. 2 show the time dependence of power, instantaneous frequency (or chirp) and frequency spectrum in both polarizations at the point where quasi-zero intensity is reached. Panel (f) shows that short-lived, very large frequency shifts (with ~3 THz amplitude, and with opposite signs for the two polarization modes) occur at the center of the ODRW. Note that the power spectrum of the high and low frequency polarization components down-shifts or up-shifts its center of mass, respectively, as shown in Fig. 2(g).

**Figure 3:**
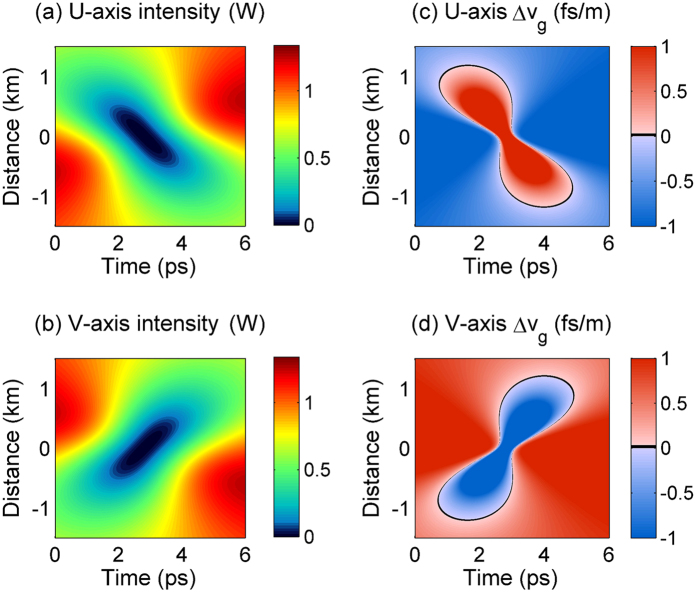
Instantaneous (or local) light velocity along the ODRW. Left column: intensity of orthogonal U and V components of ODRW solution of the vector NLSE in the normal dispersion regime for the pump power *P* = 1.9 W; Right column: corresponding plot of the instantaneous or local light velocity Δv_g_ versus propagation distance, showing that there is a localized event horizon in space-time (black curve) defined by the condition Δv_g_ = 0, where indeed light slows down (or speeds up) until it reaches a zero speed. Note that the value range of color-bars is arbitrarily restricted to the range [−1,1] to better highlight the event horizon curve.

Figure 3 provides the plot of the instantaneous (or local) light velocity Δv_g_(z,t) in each axis of polarization of the ODRW, as a function of propagation distance z. The velocity Δv_g_ is obtained as the sum of the constant linear group velocity at the frequency of each pump wave, and the local phase velocity induced by the intensity-dependent phase shift. As it can be seen, in each polarization around the notch where the light intensity is close to zero, there is a localized event horizon in space-time (black curves), where indeed light slows down (or speeds up) until it reaches the zero speed (or Δv_g_ = v_gh_ = 0). Note that the zero velocity horizons encircle the minimum intensity point at the middle of the notch.

For the U polarization, away from the ODRW the light speed is negative, and equal to its linear value Δv_g_ = −2/δ. When approaching the event horizon from the outside of the notch, the high-frequency (slow) wave with amplitude U is frequency down (or red) shifted, thus experiencing a speed boost or acceleration until it stands still as the horizon is reached (in the average group velocity reference frame). A transient black hole event horizon (a region in space time that light cannot cross) is thus formed. Similarly, for the low-frequency (fast) wave V, away from the ODRW the light speed is positive, and equal to its linear value Δv_g_ = 2/δ. As the event horizon is approached from the outside, the intensity dependent frequency up (or blue) shift leads to a light slowing down, until a white hole event horizon results, where again light stands still. Remarkably, Fig. 3 also shows that inside the intensity notch in regions encircled by the horizons, the local light speed is positive for the U polarization and negative for the V polarization. Still, as the event horizons are approached from the inside of the notch, light stops and cannot exit from the hole. See Supplementary Section for more details about the analysis of emergence of event horizons as a function of the ODRW features.

Differently with the case of bright-pulse or soliton induced event horizons^10^, here the horizons are spatiotemporal localized curves, which surround the intensity notch of the ODRW (see Fig. 3). Therefore the red and blue frequency shifts that are observed at the ODRW event horizons are of transient character, and they fully disappear after the ODRW has vanished (for better overview of the full wave kinematics, see also the spectrogram evolution for both polarization modes along propagation in the supplementary video).

### Experimental setup

**Figure 4:**
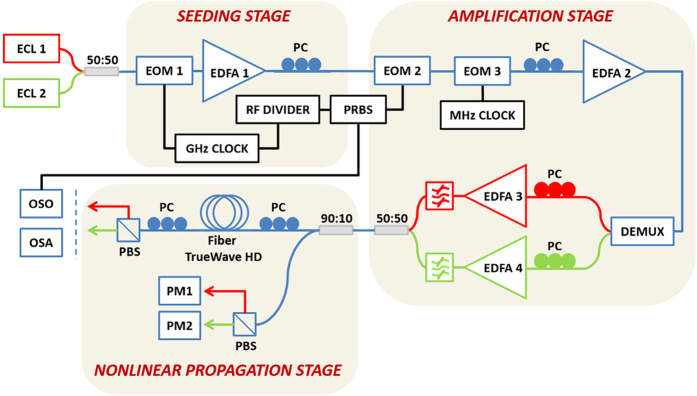
Experimental setup, red and green lines depict the two wavelength-division-multiplexed and orthogonally polarized pumps. ECL: external-cavity diode laser; 50/50 & 90:10: fiber couplers; EOM: (intensity or phase) electro-optic modulator; EDFA: Erbium doped fiber amplifier; PRBS: pseudo-random binary sequence generator; PC: polarization controller; PM: power-meter; OSO: optical sampling oscilloscope; OSA: optical spectrum analyzer.

We used the experimental configuration of Fig. 4 for creating a ODRW of light in the optical fiber. The two colliding cw pumps (red and green lines) were provided by wavelength-division-multiplexed, orthogonally polarized diode laser pumps. To induce the initial intensity perturbations, we used an electro-optic modulator (i.e., seeding stage). The orthogonal, frequency-shifted and modulated pumps were amplified and injected in a 3 km long standard telecommunication fiber with normal chromatic dispersion, and very low polarization mode dispersion. The last condition is important to guarantee the strong linear polarization mixing that is necessary to arrive to the Manakov system. For further details about the experimental setup, see the Methods section. To generate an optical ODRW, it is necessary that the input modulation breaks the stability of the orthogonal polarization pumps. The stability analysis of the Manakov system (see Supplementary Section) leads to the small-signal sideband gain shown in Fig. S1a: when the total pump power P grows larger than a certain critical value P = P_0_, the band of unstable sideband frequency shifts extends from the pump spacing down to zero, the baseband MI condition. Correspondingly, perturbations with an arbitrarily low frequency still experience exponential gain, albeit at progressively lower rates. It turns out that it is precisely such condition that enables the growth of an isolated ODRW^30^. In our experiments, the initial modulation frequency (35 GHz) was chosen to be half of the value that leads to peak PMI gain. This choice stems from the trade-off of imposing the slowest modulation on the one side, while still having sufficient sideband gain to observe the emergence of the ODRW within the 3 km fiber length. Such value of fiber length was also chosen to satisfy the requirement that fiber losses have virtually no impact on the propagation dynamics.

### Experimental results

**Figure 5:**
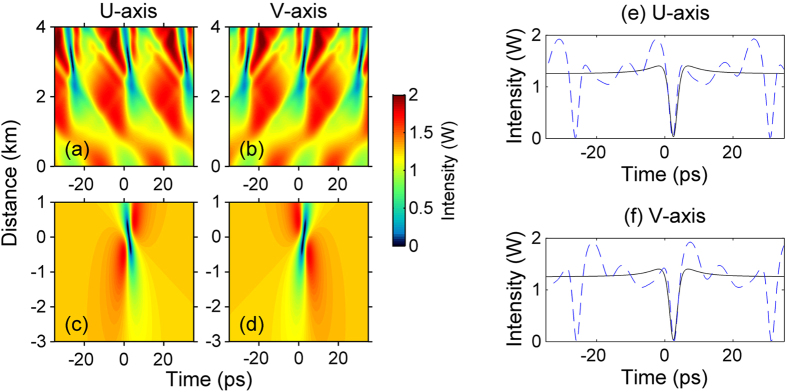
Contour plots of optical intensity in orthogonal polarization modes. (**a,b**) numerical solutions showing the generation a periodic train of optical dark rogue waves from an initially temporally modulated background (note that distance of light hole center is arbitrarily shifted at origin). (**c,d**) Exact solution of an isolated dark rogue wave. (**e,f**) Comparison of temporal intensity profiles obtained in the previous panels at the dark rogue wave centers.

**Figure 6:**
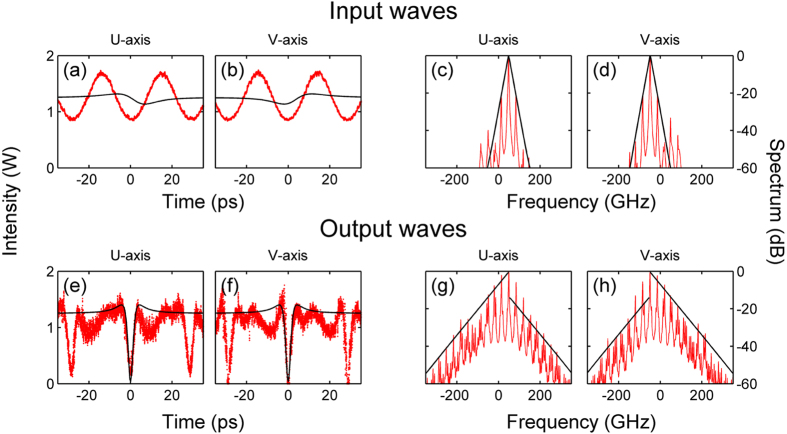
Experimental observation of the ORRW. (**a,b**) Temporal profiles of power in the U and V polarization modes at the fiber input (red solid traces). (**c,d**) Power spectra at the fiber input. (**e,f**) Output intensities after 3 km of optical fiber. (**g,h**) Power spectra at the fiber output. Solid black lines represent the analytical dark rogue wave solution.

To validate the use of the Manakov system, first we carried out an extensive analysis of spontaneous (i.e., quantum-noise induced) PMI in the fiber. Experimental spectra showing the emergence of noise sidebands, which agree quantitatively well with the predictions of the linear stability analysis, are shown in the Supplementary Section. When the modulating signal is present, the nonlinear reshaping of the counter-propagating (in a reference frame which travels with the average group velocity of the two pumps) polarizations leads to the generation of a time periodic train of black intensity notches, as shown in the numerical intensity plots of Fig. 5a,b. Quite remarkably, Fig. 5c,d show that each of the generated notches closely matches the shape of a single, isolated ODRW which corresponds to the exact solution (see also Fig. 5e,f). This is clearly confirmed by the experimental results of Fig. 6: here we compare input and output (after 3 km of optical fiber) intensities and power spectra from the experiments with an input periodic intensity modulation, and their corresponding analytical ODRW solutions. As can be seen, an overall excellent quantitative agreement (with no adjustable parameters) is obtained between theory and experiments both in the time and in the frequency domains. Only slight discrepancies appear due to the non-ideal initial conditions used for the generation of the ODRW. The observed spectral asymmetry provides a clear signature of the strong nonlinear chirping which prevents light from exiting the dark notch.

**Figure 7:**
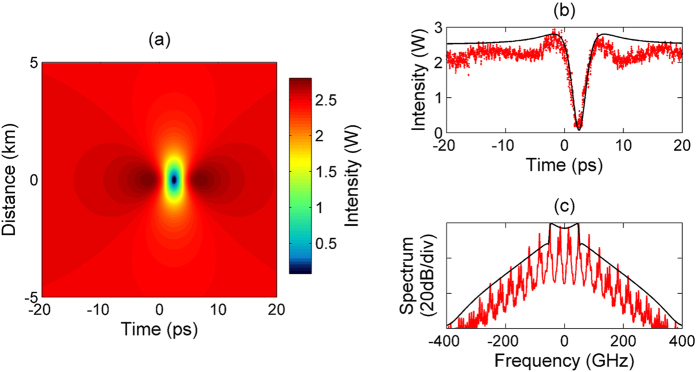
(**a**) Contour plot of the total intensity of the ODRW solution, note that distance of the dark notch center is arbitrarily shifted at the origin. (**b,c**) Comparison between analytical (black lines) and experimental results (red lines) at the optical ODRW center in both time and spectral domains, respectively.

In addition to Fig. 6 showing the comparison between experimental results and the corresponding analytical dark rogue wave solution for each of the polarization modes, Fig. 7 reports the comparison for the sum of optical intensities of both polarization modes. Panel (a) highlights the temporal symmetry during the evolution of the total intensity of the ODRW along the propagation distance, which reveals a constant average group velocity. As can be seen in panels (b) and (c), both temporal and spectral profiles of the total optical intensity at the dark rogue wave center are symmetric. The observed spectral symmetry provides a clear signature of an effective event horizon, formed by coupled black- and white-hole horizons. The sum of optical intensities of both polarization modes can be considered as the global physical picture of the dark rogue wave.

## Discussion

In summary, by using standard telecom components, we have been able to provide the first experimental observation of an optical dark rogue wave, that is, a spatio-temporal localized hole of light that becomes nearly black at its middle. This result opens the way to the challenging frontier of rogue wave research on multi-component wave systems. For example, consider two interacting Bose-Einstein condensates, which are described by a pair of Gross-Pitaevskii equations formally identical to the Manakov system (1). The two-component NLSEs (1) are also obtained in oceanography as a result of the crossing of two opposite currents, which may lead to the controlled water-tank based experimental demonstration of water dark rogue waves.

The presence of event horizons connected with the dark rogue wave suggests us to the possibility to introduce a multi-component analog model for describing the light kinematics around gravitation-induced black or white holes. The two polarization components of the ODRW could provide the optical analogs of gravitational black or white holes, originating from fiber nonlinearity-induced horizons experienced by colliding and orthogonally polarized modulated wavefronts. The light wave kinematics of the ODRW exhibits a fundamental difference with respect to the soliton induced horizons which have been investigated before. Indeed, the present black and white hole horizons are localized both in time and in space, as they are inherently linked with the transient appearance and disappearance of the ODRW, and not with a space stationary object such as an optical (bright or dark) soliton. Hence, in stark contrast with soliton-induced horizons that lead to permanent frequency conversions of probe beams, the red and blue frequency shifts that are associated with the ODRW event horizons are of purely ephemeral character: they appear from nowhere and disappear without leaving a trace, just as the rogue wave does.

Finally, note that the spontaneous creation of photon pairs induced by PMI amplification of quantum noise (as discussed in details in part 1 of the Supplementary section), which unavoidably accompanies the process of ODRW generation induced by the input signal seed, could lead to the study of the optical analog of Hawking radiation. Although the ODRW is an ephemeral wave structure, the amplification of quantum noise is not, hence Hawking radiation will persist even after the disappearance of the ODRW. The rigorous investigation of the analogy between ODRWs and gravitational black or white holes, and the generation of optical Hawking radiation, are beyond the scope of the present paper and left for further work.

## Methods

### Experiments

The two pump waves were superposed with a polarization maintaining fiber optical coupler with a 50:50 coupling ratio. An intensity modulator (EOM1) driven by a 35-GHz RF clock was used to generate sidebands on either side of both pumps. An erbium-doped fiber amplifier (EDFA1) was used to compensate the insertion loss introduced by the electro-optic modulator. The sinusoidal perturbation on each pump with adjustable frequency and amplitude then seeds the PMI process. Here the perturbation frequency was chosen to be half the peak PMI gain for the following two-pump system: 100 GHz-frequency spacing and 2.5 W total input power. The perturbation amplitude of the continuous-wave field was set to *ε* = 0.16 in order to observe the ODRW at precisely 3-km in our fiber with the available pump power. In order to reach the required peak power in our experiments, we temporally carved both pump waves thanks to an electro-optic modulator (EOM2) driven by a pseudo-random binary sequence generator. More precisely, 100-ns square pulse trains were generated with a duty cycle of 1:10 and then amplified (EDFA2). Such pulses provide a quasi-cw background condition, since the pulse duration is about four orders of magnitude larger than both the spontaneous and the induced modulation period. Moreover, to suppress stimulated Brillouin scattering (SBS) that may occur in the optical fiber, a phase modulator (EOM3) was inserted into the setup in order to increase the spectral linewidth of the two pump waves. The phase modulator was driven by a 67-MHz RF signal, thus enabling us to work at relatively high pump powers, still being far below the SBS threshold. The two pump waves were spectrally separated by means of a programmable optical filter (DEMUX). A pair of polarization controllers (PCs) allowed us to obtain two pumps with orthogonal linear states of polarization. The pumps were finally recombined after their independent amplification by high power erbium-doped fiber amplifiers (EDFA3&4) and before injection into the optical fiber. The optical fiber used in our experiment was a reverse-TrueWave fiber with the chromatic dispersion of −14 ps/nm/km, the nonlinear coefficient γ = 2.4 W^−1^.km^−1^ and the attenuation of 0.25 dB/km at λ_0_ = 1554.7 nm. This fiber has a very low PMD equal to 0.017 ps.km^−1/2^. Two fiber spans were used in our experiments. A 3 km-long span was used to generate the ODRW (main manuscript). A longer span of 5 km was also used to validate the use of the Manakov model by analyzing the spontaneous PMI (supplementary information). At the fiber output, a polarization beam splitter (PBS) selects the output light propagating in the two orthogonal linear polarizations. The output light was simultaneously analyzed both in the spectral and temporal domain by means of an optical spectrum analyzer (OSA) and an optical sampling oscilloscope (OSO). Spectral measurements (Yokogawa - AQ6370 OSA) were carried out with 0.02 nm resolution bandwidth. The optical sampling oscilloscope (Picosolve PSO-101) had 0.8 ps resolution. The orthogonal polarization states were continuously tracked before injection into the fiber thanks to power measurements after their separation into a polarization beam splitter. We performed cut-back measurements (not shown here) to verify the kinematics of the ODRW generation and disappearance at distances before and after the ODRW centre point (3 km).

### Theoretical solution and numerical simulation

The ODRW solution of the Manakov system that is presented in the Supplementary Section is depicted in Figs 2 and 3 by using a total pump power *P* = 1.9 W, a pump spacing equal to 100 GHz, and the fiber parameters given above. Figures 5, 6, 7 and S2 report a similar theoretical solution but using the pump power used in the experiments (*P* = 2.5 W). The Manakov equations were numerically solved by using the split-step Fourier method^26^. The simulation shown in Fig. 5 considered the experimental input conditions, namely, a weakly modulated continuous-wave field with modulation frequency and depth corresponding to our experiment. The numerical results in Fig. S1b,c also included fiber loss and a −70 dB noise background.

## Additional Information

**How to cite this article**: Frisquet, B. *et al*. Optical Dark Rogue Wave. *Sci. Rep.* **6**, 20785; doi: 10.1038/srep20785 (2016).

## Supplementary Material

Supplementary Information

Supplementary movie1
