# Supplementary material for: Optical Dark Rogue Wave
Source: Sci Rep. 2016 Feb 11;6:20785. doi: 10.1038/srep20785 (PMC4749966; doi:10.1038/srep20785)
Supplement: Supplementary Information [file srep20785-s1.pdf]

Supplementary information to Optical Dark Rogue Wave

Benoit Frisquet[1], Bertrand Kibler[1*], Philippe Morin[1], Fabio Baronio[2], Matteo Conforti[3], Guy Millot[1], and Stefan Wabnitz[2]

[1] *Laboratoire Interdisciplinaire Carnot de Bourgogne (ICB), UMR 6303 CNRS - Université de Bourgogne, F-21078 Dijon, France*

[2] *Department of Information Engineering, Università di Brescia, and INO-CNR, Brescia, Italy*

[3] *PhLAM/IRCICA UMR 8523/USR 3380 CNRS - Université de Lille 1, F-59655 Villeneuve d'Ascq, France*

*bertrand.kibler@u-bourgogne.fr

1. Manakov system: Linear stability analysis and polarization modulation instability

The propagation of two orthogonally polarized optical pump waves with a relative frequency offset, say, $\Delta = \Delta\omega/2\pi$, in the normal dispersion regime of a randomly birefringent telecom fiber with relatively low polarization mode dispersion is described in terms of two incoherently coupled Nonlinear Schrödinger equations (CNLSEs) or Manakov system [S1-S3]:

$$\begin{aligned} i\frac{\partial U}{\partial z} + i\frac{\delta}{2}\frac{\partial U}{\partial t} - \frac{\beta_2}{2}\frac{\partial^2 U}{\partial t^2} + \gamma(|U|^2 + |V|^2)U = 0 \\ i\frac{\partial V}{\partial z} - i\frac{\delta}{2}\frac{\partial V}{\partial t} - \frac{\beta_2}{2}\frac{\partial^2 V}{\partial t^2} + \gamma(|U|^2 + |V|^2)V = 0 \end{aligned} \qquad \text{(s1)}$$

Here z and t denote the propagation distance and retarded time (in the frame travelling at the group-velocity evaluated at the central carrier frequency ω_o) coordinates; U and V are the complex slowly varying amplitudes of the two pump waves at frequencies $\omega_{u,v} = \omega_o \pm \pi\Delta$, respectively, and δ is associated with their group-velocity mismatch (GVM) owing to normal group-velocity dispersion. In fact, the U (V) pump is a slow (fast) wave with respect to the waves at the carrier frequency ω_o. β_2 and γ are the group-velocity dispersion and the effective Kerr nonlinear coefficient at frequency ω_o. Note that in Eqs. (s1) the variations of β_2 and γ as a function of the frequency are neglected for the frequency detuning that is considered in our experiments ($\Delta \leq 500$ GHz). The GVM parameter is then simply defined as $\delta = \beta_2\Delta$, whereas the nonlinear coefficient is written as $\gamma = \frac{8n_2\omega_0}{9cA_{eff}}$, where c designates the light velocity, $n_2 = 2.6\ 10^{-20}$ m^2/W is the nonlinear index coefficient, and A_{eff} designates the effective core area at the carrier angular frequency ω_o. The factor 8/9 takes into account random variations of the intrinsic fiber birefringence [S3]. We would now like to emphasize the fact that coherent coupling terms [S3] have been neglected in Eqs. (s1), since they only play a role in a very small parameter range (small values of Δ) which is outside the operating conditions considered in our experimental study. On the other hand, we would like to mention that Raman effects are not considered in Eqs. (s1) since our experimental conditions (relatively low input power and short fiber length) were selected for these effects to be negligible. The accuracy of Eqs. (s1) in representing MI effects of polarized waves in a randomly birefringent, low-PMD fiber was recently confirmed in the anomalous GVD regime [S4].

The linear stability analysis of the steady state solution of Eqs. (s1) leads to the eigenvalue equation $[M][Y] = K[Y]$, with the eigenvector defined as $[Y]^T = [U_a, U_s^*, V_a, V_s^*]$, where U_s and U_a are the amplitudes of the Stokes and anti-Stokes sidebands for the pump wave of amplitude U, respectively, whereas v_s and v_a represent the Stokes and anti-Stokes sidebands for the pump wave of amplitude V, respectively. Throughout this work we assume that the power P injected into the fiber is equally

distributed between the two pumps, i.e. $|U(z=0,t)|^2 = |V(z=0,t)|^2 = P/2$. The stability matrix of the system $[M]$ is then defined as:

$$[M] = \begin{bmatrix} A & B \\ B & C \end{bmatrix} \tag{s2}$$

$$A = \begin{bmatrix} -d + b + \frac{g}{2} - k & \frac{g}{2} \\ -\frac{g}{2} & -d - b - \frac{g}{2} - k \end{bmatrix}$$

$$B = \begin{bmatrix} g/2 & g/2 \\ -g/2 & -g/2 \end{bmatrix} \qquad C = \begin{bmatrix} d + b + \frac{g}{2} - k & g/2 \\ -g/2 & d - b - \frac{g}{2} - k \end{bmatrix}$$

with $d = \pi\Delta\beta_2\Omega$, $b = \frac{\beta_2\Omega^2}{2}$ and $g = \gamma P$, where Ω is the angular frequency of the perturbation related to the wave number K by the dispersion relation $det([M] - K[I]) = 0$. The MI phenomenon occurs when the wave number K of the perturbation possesses a nonzero imaginary part, and manifests itself in an exponential increase of the amplitude of the perturbation, whose importance is measured by a power gain G defined by $G(\Omega) = 2|Im(K)|$. Thus, one obtains the following dispersion relation:

$$K^2 = d^2 + b^2 + bg - \sqrt{b(4d^2(b+g) + bg^2)} \tag{s3}$$

which yields the condition for polarization MI to occur, that is,

$$max\left[0, \left(\Delta^2 - \frac{g}{\pi^2\beta_2}\right)\right] \leq \left(\frac{\Omega}{2\pi}\right)^2 \leq \Delta^2 \tag{s4}$$

Figure S1(a) shows the MI gain spectrum for different pump power values, obtained for our fiber parameters at λ_0 = 1554.7 nm: $\boldsymbol{\beta_2}$ = 18 ps^2 km^{-1} (or D = -14 ps/nm/km), and γ=2.4W^{-1}.km^{-1}. As can be seen, whatever the input power, the high cutoff frequency is fixed and remains equal to the pump spacing, whereas the low cutoff frequency depends on the input power level. $\boldsymbol{P_0} = \frac{9\pi^2\Delta^2\beta_2}{8\gamma}$ denotes the power for which the low cutoff frequency vanishes (e.g. $\boldsymbol{P_o}$=0.83W for Δ=100 GHz). $\boldsymbol{P_0}$ defines the frontier between two different regimes of PMI gain, namely, passband and baseband regimes. It was recently pointed out that the existence of isolated rogue wave solutions can be associated with the presence of baseband PMI (i.e., $\boldsymbol{P} \geq \boldsymbol{P_0}$) [S5,S6]. A remarkable issue is that there is no critical power beyond which PMI disappears, but there is a monotonic increase of the peak gain with input power, and a gain saturation as the power grows above about 10 $\boldsymbol{P_0}$ (see Fig. S1a). The asymptotic solutions to the dispersion relation (Eq. (s3)) that are found in the limit of large values of $\boldsymbol{g}$ lead to the saturation values of the peak gain $\boldsymbol{G_{sat}} = \boldsymbol{2\pi^2\Delta^2\beta_2}$ and optimum modulation frequency $\boldsymbol{f_{sat}} = \boldsymbol{\Delta/\sqrt{2}}$.

To validate the use of the Manakov system, we carried out an extensive analysis of spontaneous (i.e., quantum-noise induced) PMI in the fiber. We present some of the experimental results in Fig. S1b-c, in particular when considering the following input parameters (P = 1.69 W for Δ=200 GHz). The red solid line indicates the experimental spectrum, showing the emergence of noise sidebands that agree well with the predictions of the linear stability analysis of panel (a) (black line) for frequency position of MI gain bands. The corresponding numerical simulation of Manakov system (Eqs. (s1)) is also depicted (dashed blue line) and fits well the experimental observation of PMI gain bands. Note that the experimental setup to observe Manakov PMI in the normal dispersion regime of a telecom fiber span is the same that the one used for the observation of ODRW, except that the optical seeding stage is removed, and the fiber length is now 5 km.

Figure S1d compares the observed cumulated (over the entire fiber span of 5 km) peak PMI gain with the analytical predictions, for different initial frequency separations among the pump waves. The PMI gain grows larger with the pump spacing, however at large frequency separations the available gain is reduced with respect to the small signal analytical predictions (which neglect pump depletion), in excellent agreement with the full numerical solution of the Manakov system. Note that choosing non-orthogonal input polarization conditions for the pump waves completely prevents the emergence of any PMI gain bands in our experiments.

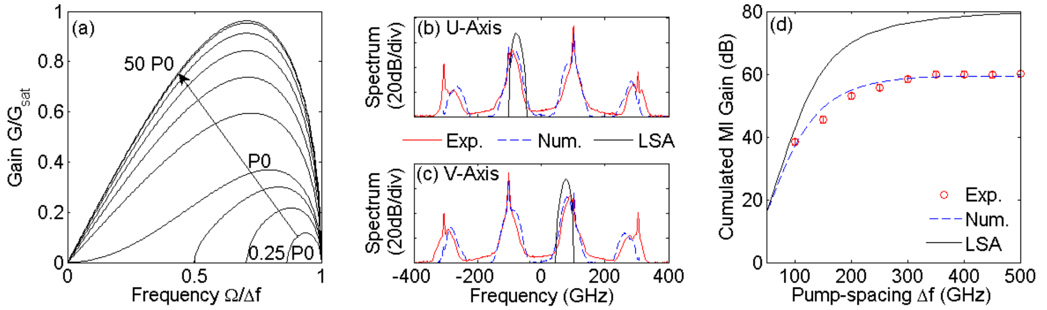

Figure S1 | **a**, Variation of PMI gain *G* for each pump wave as a function of the input pump power: as *P* is equal or greater than P_0, a baseband PMI is obtained. **b,c**, Input and output spectrum: red solid curves indicate the experimental spectrum. Solid blue curves are obtained from the numerical solution of the vector NLSE, and black curves represent the theoretical gain shown in panel **a**. **d**, Variation of PMI gain with pump spacing from the analytical gain of panel a (solid black line), from the numerical solution Manakov equations (dashed blue line) , and from the experiments (empty red dots).

2. Manakov system: Dark rogue wave solution

Equations (s1) exhibit a multi-component solution, with the property of describing unique wave events, i.e. "dark rogue waves" isolated in space and time. This solution exists in the defocusing regime of the vector NLSE, in the subset of the parameter space where baseband PMI is present [S5]. The ODRW solution which is written in terms of rational functions of coordinates, can be expressed as:

$$U_{bh} = \sqrt{\frac{2}{\gamma}} a e^{i4za^2} \frac{\alpha_u\sqrt{2\beta_2}pt + 2p^2t^2 + \beta_2\beta\theta_u + \sqrt{2\beta_2}\beta pt\theta_u + i\alpha_u\beta_2 p^2 z + \beta_2 p^4 z^2}{\sqrt{2\beta_2}\beta pt + 2p^2t^2 + \beta_2(\beta + p^4z^2)}$$

$$V_{bh} = \sqrt{\frac{2}{\gamma}} a e^{i4za^2} \frac{\alpha_v\sqrt{2\beta_2}pt + 2p^2t^2 + \beta_2\beta\theta_v + \sqrt{2\beta_2}\beta pt\theta_v + i\alpha_v\beta_2 p^2 z + \beta_2 p^4 z^2}{\sqrt{2\beta_2}\beta pt + 2p^2t^2 + \beta_2(\beta + p^4z^2)} \quad \text{(S5)}$$

with

$$a = \sqrt{\gamma P/4}\,,\ q = -\delta/\left(2\sqrt{2\beta_2}\right),$$
$$\theta_n = (2q_n + ip)/(2q_n - ip)\ ,\ \alpha_n = \alpha = 4p^2/(p^2 + 4q^2) \text{ where } n = u, v$$
$$q_u = -q\,,\ q_v = q\,,\ \beta = p^3/\left(\chi(p^2 - 4q^2)\right)\ ,\ \chi = Im(k)\,,\ p = 2Im(\lambda + k)$$

λ is the double solution of the polynomial $\lambda^3 + A_2\lambda^2 + A_1\lambda + A_0 = 0$, with $A_0 = -k^3 + k(q^2 + 2a^2)$; $A_1 = -k^2 - q^2 + 2a^2$; $A_2 = k$. The constraint on the double roots is satisfied when the discriminant is zero, which results in the fourth order polynomial condition:
$k^4 + D_3k^3 + D_2k^2 + D_1k + D_0 = 0$,
with $D_0 = (q^2 - 2a^2)^3/(16q^2)$; $D_1 = 0$; $D_2 = (a^2 - 10a^2q^2 - 2q^4)/(4q^2)$; $D_3 = 0$.

For example, for the parameters involved in Fig. 2 (main manuscript), $\beta_2 = 18$ ps^2 km^{-1}, γ=2.4W^{-1} km^{-1}, δ =1.8 ps km^{-1}, and P = 1.9 W, we obtain the following truncated results (by choosing a complex value of k): $a =$ 1.067708, $k = 0.251791$i, $\lambda = -0.785421$i, $q = -0.942478$.

3. Polarization rogue wave and event horizons

As shown in Fig. 3, the ODRW induces the emergence of localized event horizons in space-time, in particular for each polarization around the notch where the light intensity is close to zero. As the event horizons originate from the intensity-dependent phase shift, we carefully checked the impact of input power P on the features of localized event horizons. For the experimental parameters that we used, a power threshold was revealed (P_{th} = 0.98 W) to observe the emergence of localized event horizons in space-time. Below P_{th}, the local phase velocity induced by the intensity-dependent phase shift is too small to counteract the constant linear group velocity and reach the zero speed (or $\Delta v_g = v_{gh} = 0$). Beyond P_{th}, there is a localized event horizon in space-time, where indeed light slows down (or speeds up) until it reaches the zero speed (see Fig. 3). Note that the zero velocity horizons encircle the minimum intensity point at the middle of the notch, and its localization is strongly related to the notch profile in space-time, which evolves with the input power. Indeed, when increasing the input power beyond 1.98 W, the light intensity in each polarization dips to exactly zero (only once or twice for P > 1.98 W): as a consequence, in each polarization component there is a double loop event horizon appearing in space time (see Fig. S2), where indeed light reaches the zero speed. When carefully examining the evolving intensity dip in space-time between the two zero intensity points, we note that it slightly varies from 0 to 0.03 and then it goes back to 0 W. Such small intensity variations cannot be revealed experimentally (less than 2% variations compared to the maximal peak intensity of the wave profile), so that the presence of complex double-loop event-horizon dynamics cannot be directly confirmed by our experiments. Note that the experimental input power (P = 2.5 W) was chosen in such a way that we observe the middle point of the ODRW for the shortest fiber span (z = 3 km), in order to overcome the detrimental impact of fiber losses on the dynamics associated with the lossless Manakov model. Both temporal and spectral features presented in Fig. 6 allow to confirm the opening and closing of localized event horizons for each polarization (as shown in Fig. 3), even though more complex dynamics remain hidden in the center of the ODRW.

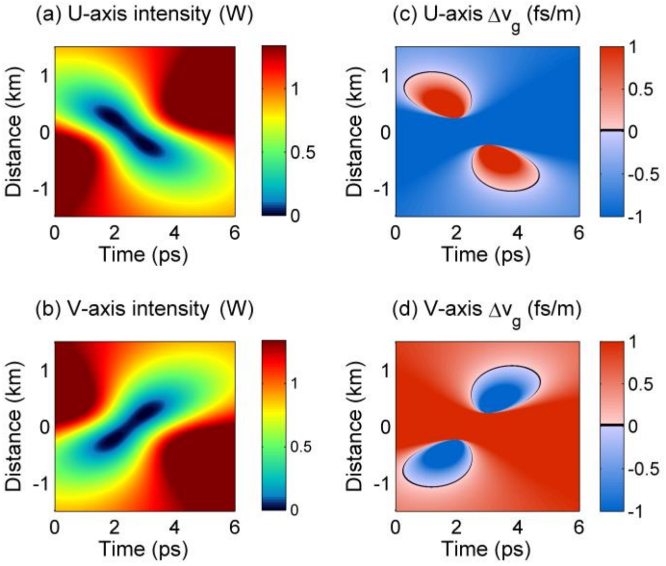

Figure S2 | Left column: intensity of orthogonal U and V components of ODRW solution of the vector NLSE in the normal dispersion regime for the experimental pump power *P* = 2.5 W; Right column: corresponding plot of the instantaneous or local light velocity Δv_g versus propagation distance, showing that there is a localized event horizon in space time (black curve) defined by the condition Δv_g=0, where indeed light reaches the zero speed. Note that the value range of color-bars is arbitrarily restricted to the range [-1,1] to better highlight the event horizon curve.

References for supplementary information

[S1] S. V. Manakov, "On the theory of two dimensional stationary self-focusing of electromagnetic waves," *Sov. Phys. JETP* **38**, 248-253 (1974).

[S2] P. K. A. Wai and C. R. Menyuk, "Polarization mode dispersion, decorrelation, and diffusion in optical fibers with randomly varying birefringence," *J. Lightwave Technol.* **14**, 148-157 (1996).

[S3] D. Marcuse, C. R. Menyuk, and P. K. A. Wai, "Application of the Manakov-PMD equation to studies of signal propagation in optical fibers with randomly varying birefringence," *J. Lightwave Technol.* **15**, 1735-1746 (1997).

[S4] J. Fatome, I. El-Mansouri, J. L. Blanchet, S. Pitois, G. Millot, S. Trillo, and S. Wabnitz, "Even harmonic pulse train generation by cross-polarization-modulation seeded instability in optical fibers," *J. Opt. Soc. Am. B* **30**, 99-106 (2013).

[S5] F. Baronio, M. Conforti, A. Degasperis, S. Lombardo, M. Onorato, and S. Wabnitz, "Vector rogue waves and baseband modulation instability in the defocusing regime," *Phys. Rev. Lett.* **113**, 034101 (2014).

[S6] F. Baronio, S. Chen, P. Grelu, S. Wabnitz, and M. Conforti "Baseband modulation instability as the origin of rogue waves," *Phys. Rev. A* **91**, 033804 (2015).
